# Using process features to investigate scientific problem-solving in large-scale assessments

**DOI:** 10.3389/fpsyg.2023.1131019

**Published:** 2023-04-18

**Authors:** Tao Gong, Lan Shuai, Yang Jiang, Burcu Arslan

**Affiliations:** ^1^School of Foreign Languages, Zhejiang University of Finance and Economics, Hangzhou, Zhejiang, China; ^2^Educational Testing Service, Princeton, NJ, United States; ^3^Google, New York, NY, United States

**Keywords:** scientific problem solving, fair test, exhaustive test, preparation time, execution time

## Abstract

**Introduction:**

This study investigates the process data from scientific inquiry tasks of *fair tests* [requiring test-takers to manipulate a target variable while keeping other(s) constant] and *exhaustive tests* (requiring test-takers to construct all combinations of given variables) in the National Assessment of Educational Progress program.

**Methods:**

We identify significant associations between item scores and temporal features of preparation time, execution time, and mean execution time.

**Results:**

Reflecting, respectively, durations of action planning and execution, and execution efficiency, these process features quantitatively differentiate the high- and low-performing students: in the fair tests, high-performing students tended to exhibit shorter execution time than low-performing ones, but in the exhaustive tests, they showed longer execution time; and in both types of tests, high-performing students had shorter mean execution time than low-performing ones.

**Discussion:**

This study enriches process features reflecting scientific problem-solving process and competence and sheds important light on how to improve performance in large-scale, online delivered scientific inquiry tasks.

## 1. Introduction

The past two decades have witnessed an increasing use of computers and relevant technologies in classroom teaching and learning (Hoyles and Noss, 2003) and a swift transition from traditional paper-and-pencil tests to *digitally-based assessments* (DBAs) (Zenisky and Sireci, 2002; Scalise and Gifford, 2006) that accommodate advancement of educational technologies. Along with these trends, the National Assessment of Educational Progress (NAEP)^1^ began to use hand-held tablets to administer math assessments in the U.S. in 2017, so did other disciplines afterward. Capable of recording multi-dimensional data, DBAs offer ample opportunities to systematically investigate U.S. students’ problem-solving processes through well-designed *technology-enhanced items* (TEIs) (National Assessment Governing Board, 2015). TEIs refer broadly to computer-aided items that incorporate technology beyond simple option selections as test-takers’ response method (Koedinger and Corbett, 2006). In a TEI, test-takers are asked to interact with computers by performing a series of actions to solve one (or multiple) problem. For example, in scientific inquiry TEIs of *fair tests* (Chen and Klahr, 1999), students are asked to adjust a target variable in an experimental setting or condition while keeping other(s) constant, to reveal effect or outcome of the target variable. In another type of scientific inquiry TEIs, *exhaustive tests* (Montgomery, 2000; Black, 2007), students are required to construct all possible combinations of given variables to investigate what combination(s) leads to a specific outcome (see Section 2 for details). In both types of tests, students need to apply the *control-of-variables strategy* (CVS, see Section 2 for details), a domain-general processing skill to design controlled experiments in a multi-variable system (Kuhn and Dean, 2005; Kuhn, 2007).

Beyond final responses, interactive actions of students are captured as *process data*.^2^ Such data help (re)construct problem-solving processes, reflect durations (or frequencies) of major problem-solving stages, and infer how students deploy strategies they seem to know (Pedaste et al., 2015; Provasnik, 2021), all of which provide additional clues of students’ problem-solving behaviors (Kim et al., 2007; Ebenezer et al., 2011; Gobert et al., 2012). For example, in drag-and-drop (D&D) items, a popular type of TEIs, students drag some objects from source locations and drop them into target positions on screen. Compared to conventional multiple-choice items, such items can better represent construct-relevant skills, strengthen measurement, improve engagement/motivation of test-takers, and reduce interference of random guessing (Bryant, 2017; Arslan et al., 2020).

Despite the advantages, process data have long been treated as by-products in educational assessments. Until recently, scholars have begun to investigate whether (and if so, how) process data inform (meta)cognitive processes and students’ strategies during problem solving (Guo et al., 2019; Tang et al., 2019; Gong et al., 2021, 2022). By reviewing pioneering studies on NAEP process data before its formal transition to DBA, Bergner and von Davier (2019) proposed a hierarchical framework that divides process data use into five levels based on its relative importance to outcome: *Level 1*, process data are irrelevant/ignored and only response data are considered; *Level 2*, process data are incorporated as *auxiliary* to understanding outcome; *Level 3*, process data are incorporated as *essential* to understanding outcome; *Level 4*, process data are outcome and incorporated into scoring rubrics; and *Level 5*, process data are outcome and incorporated into measurement models.

Most published process data studies remain up to level 2 of this framework; they directly use students’ actions, action sequences, and (partial/rough) durations of answering processes to interpret item outcome (e.g., answer change behaviors, Liu et al., 2015; response time, Lee and Jia, 2014; or action sequences, Han et al., 2019; Ulitzsch et al., 2021). Before explicitly revealing correlations between process data and individual performance, inferences from these studies remain *auxiliary* rather than *essential*. In other words, discovering process features and their relatedness to test-takers’ performance is a *prerequisite* for using process features to understand or interpret individual performance, thus reaching higher levels of the framework.

This study aims to fulfill this prerequisite by investigating process data from *scientific inquiry tasks* (see Supplementary materials) and related research questions therein in a three-step procedure:

*Define time-related features to illustrate action planning and executing stages of scientific problem solving*. Many early studies have examined action-related features that reflect conceptual formation (Jonassen, 2000; Lesh and Harel, 2011), response strategies, and internal (individual dispositions) or external (testing circumstances) factors probably affecting students’ choices of strategies (Griffiths et al., 2015; Lieder and Griffiths, 2017; Moon et al., 2018). However, the time needed for problem solving has been largely undervalued (Dostál, 2015). As an informative indicator of problem solving stages, temporal information helps characterize patterns of students, and infer (meta)cognitive processes occurring at various stages of problem solving.

**Figure 1:**
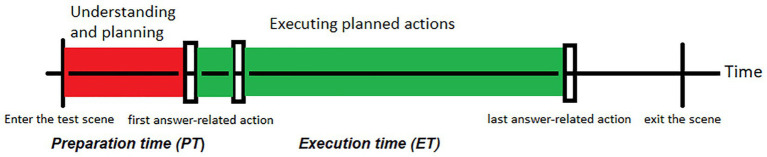
Proposed process features [preparation time (PT) and execution time (ET)] and corresponding major stages of scientific problem solving (understanding and planning, and executing planned actions, denoted by colored bars) in a scientific inquiry test item. Vertical lines denote the times when a test-taker enters and exits the task, vertical bars denote answer-related actions. See Supplementary materials for review of scientific problem solving processes.

We propose three temporal features to reflect, respectively, the major stages of scientific problem solving (see Figure 1). In an assessment setting, *preparation time* (*PT*) is defined as the time difference (duration) between the moment students enter a test scene and when they make their first answer-related event. It denotes the duration while students understand instructions and conceptually plan their actions, before making any. *Execution time* (*ET*) is defined as the time difference between students’ first and last answer-related events. It measures the duration while students execute their planned actions. *Mean execution time per* (*MET*) is measured as *ET* divided by the number of answer related events.^3^ *ET* reflects total efforts of students casted to construct their answers, including setting up answers and revising or (possibly) reviewing their choices, whereas *MET* reflects average effort over total events. Controlling for answer-related events, *MET* indicates the efficiency of action execution. Our study examines whether these temporal features significantly correlate item scores and characterize high/low-performing students in test scenes.^4^

*Explore correlations between process features and item scores*. This is the missing link in many existing studies of problem solving; some cannot verify such correlations, since the categorical features (e.g., action sequences) used cannot fit for correlation tests, whereas others, directly assuming such correlations, skip this step and use process features to inform/interpret performance. Neither approach is complete. Our study focuses on detecting correlations between continuous process features and item scores and explaining feature differences across score groups.

We apply two statistical tests to detect correlations. First, the Kruskal-Wallis test (Kruskal and Wallis, 1952) compares process features across score groups, and reports whether (at least) one of the multiple samples is significantly distinct from others. As a non-parametric version of ANOVA, this test does not require a normal distribution of the residual values of features. Extended from the Mann–Whitney test, this test is an omnibus test applicable to small-scale, independent samples from multiple groups. Second, we conduct the omnibus ANOVA between score groups, and log-transformed (base *e*) the process features to meet the normality assumption. This test is applicable to large-scale datasets. We use both methods to cross-validate obtained results by each method.

*Use process features to characterize performance (or competence) differences between students and/or tasks*. After verifying correlations between process features and item scores, we further investigate: (a) *whether there exist differences (or similarities) in the process features across score groups and/or inquiry tasks*; and (b) *whether the observed differences (or similarities) characterize problem solving performance (or competence) between high- and low-performing students and between inquiry tasks.* Answers to these questions further foster these features as informative indicators of students’ performance and pave the way for incorporating them into scoring rubrics and measurement models aiming to classify and interpret students’ behaviors.

In the following sections, we first review the CVS strategies and scientific inquiry tasks, and then define the process metrics and analysis plans. After reporting the analysis results, we answer the abovementioned questions, summarize our contributions to scientific inquiry and problem solving research, and point out the general procedure of process data use in educational assessments.

## 2. Control-of-variables strategies and scientific inquiry tasks

*Control-of-variables strategy* (CVS)^5^ has been widely studied in science assessments. CVS refers to the skill used to design controlled experiments in a multi-variable system. To avoid confounded experiments, all variables but those under investigation must be controlled in a way to meet task requirements. In the *Next Generation Science Standards* (NGSS), CVS and multivariate reasoning are viewed as two key scientific thinking skills. Central to early science instruction (Klahr and Nigam, 2004) (around grades 4–8), CVS cannot develop routinely without practice or instruction (Schwichow et al., 2016), making it a critical issue in development of scientific thinking (Kuhn and Dean, 2005). Children, adolescents, and adults with low science inquiry skills show difficulty in applying CVS in scientific problem solving (Chen and Klahr, 1999).

In large-scale assessments like NAEP, CVS is often assessed by two types of scientific inquiry tasks: fair tests and exhaustive tests. *A fair test* (see examples in Section 3.1) refers to a controlled investigation carried out to answer a scientific question about the effect of a target variable. To control for confounding factors and be scientifically sound, students are expected to apply the CVS to meet the fair test requirement that: (a) all other variable(s) are kept constant; and (b) only the target one(s) changes across conditional sets for comparison. In such a “fair” setting, the effect of the target variable(s) can be observed and less interfered by other variables. To properly complete the task, students need to choose, among possible combinations of different levels of the target and other variables, one (or a few) condition that meets the requirement. There are studies of CVS in scientific inquiry using small-scale participants and response/survey data (Kuhn, 2007). A recent meta-analysis of intervention studies (partially) designed to enhance CVS skills revealed that instruction/intervention (e.g., cognitive conflict and demonstration) influences achievement in scientific inquiry tasks (Schwichow et al., 2016).

*An exhaustive test* (a.k.a. *all-pair* or *combinatorial test*) (see examples in Section 3.2) requires test-takers to construct, physically or mentally, (nearly) all possible combinations of given variables to address an inquiry of what condition(s) induces a specific outcome. Similar to fair tests, students in exhaustive tests need to control the given variables by setting up combinations exhaustively or nearly so (in an open-ended case). Though not explicitly mentioned in NGSS, exhaustive testing is essentially related to CVS or at least a case of multivariate reasoning. How to conduct exhaustive tests is usually taught and learned relatively late in science education (around grades 9–12). Such tests have also been adopted in other fields than educational assessments, e.g., software engineering and business (Grindal et al., 2005).

## 3. Materials and methods

Our study makes use of the 2018 NAEP science pilot tasks (see Supplementary materials). It adopted four tests, respectively, from four tasks in the repertoire: two fair tests administered on fourth- and eighth-graders, respectively (the primary and middle school bands, per NGSS), and two exhaustive tests on twelfth-graders (the high school band). Table 1 shows the samples of these tests.

**Table 1:** Basic information of the testlets investigated in this paper.

Test item	Subfield	Grade	No. Students (Female, Male) for analyses
Fair test 1	Earth/space science	8	1,607 (800, 807)
Fair test 2	Physical science	4	1,990 (977, 1,013)
Exhaustive test 1	Life science	12	2,726 (1,285, 1,341)
Exhaustive test 2	Earth/space science	12	2,947 (1,465, 1,482)

Due to various reasons (e.g., early quit or data capture glitches), data of some students were missing. The rightmost column records the number of students whose process and response data were used for analyses.

Two criteria lead to the choice of these tasks. First, the sampled tests should cover most science subfields and grades in the NAEP sample. However, given that lower grade students have not been taught to solve exhaustive tasks, no such tests were administered on fourth-graders. Second, since fair tests were administered mostly on eighth-graders and exhaustive tests on twelfth-graders, it is impossible to select fair tests and exhaustive tests administered on students of the same grade. Nonetheless, since all the NAEP fair and exhaustive tests were designed by content experts following similar constructs and the only difference was that each task fell into one of the science disciplines (physical, life, earth/space sciences), the chosen tests in our study are representative.

### 3.1. The fair tests and scoring rubrics

The fair test 1 came from an earth/space science task. Its cover task^6^ is as follows. A city near a mountain suffers from strong north wind each year. The government plans to test the wind-blocking effect of three types of trees. Each type can be planted at the foot (low), side (medium), or peak (high) of the northern ridge of the mountain to reduce wind speed, and there is no interaction between tree type and mountain position (e.g., there is no preference for one type of trees to be planted at a specific position).

The whole task is presented to students through multiple scenes, some involving items. The first few scenes help students understand, represent, and explore relevant issues. After them comes the fair test scene, in which students are asked to design a controlled experiment to investigate the wind-blocking effects of the three types of trees. The follow-up scenes ask them to interpret/revisit their answers and apply their knowledge in novel conditions. Students went through these scenes in the same order and could not freely jump around.

**Figure 2:**
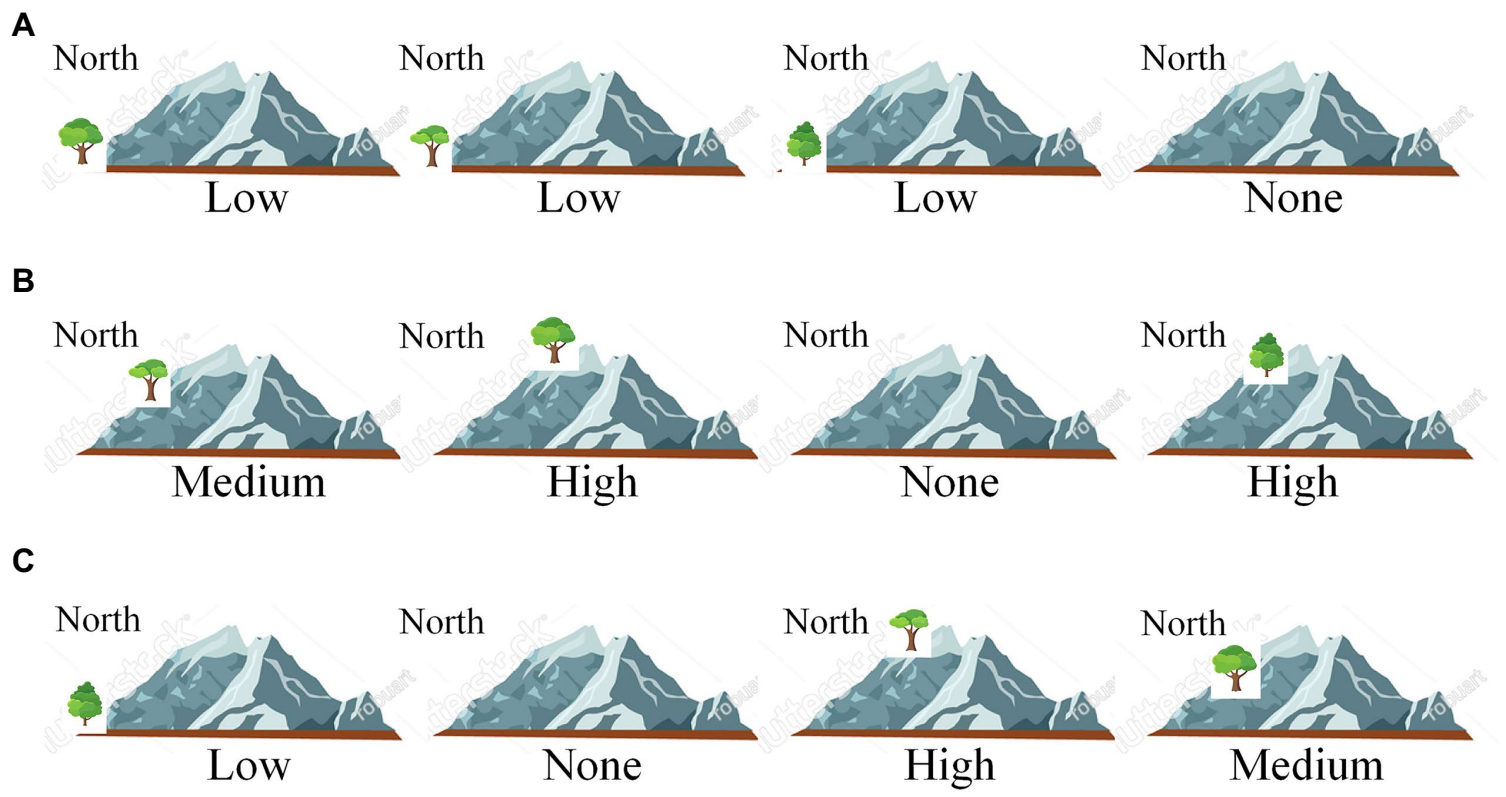
Example answers of the fair test 1. “Low,” “Medium,” “High” denote tree positions (foot, side, peak) in the northern ridge of a virtual mountain. “None” means no tree planted. In **(A)**, the first “Low” indicates that one type of trees is planted at the foot of the mountain, the second and third “Low” indicate that the other two types of trees are planted on the second and third mountains, and “None” means that the fourth mountain has no trees planted. Since the scoring rubric (see Table 2) does not specify tree type and ignores the mountain without trees, submitted answers can be simply denoted by the tree positions in the mountains with trees. In this way, answer **(A)** can be denoted as “Low; Low; Low”, answer **(B)** as “Medium; High; High”, and answer (C) as “Low, High, Medium”.

In the fair test scene (see Figure 2), students need to drag each type of the trees and drop it at one of the four virtual mountains resembling the real one near the city; students can drop the trees at the foot (low), side (middle), or peak (high) of the northern ridge of the mountain. Each mountain can hold one type of the trees, and each type can only be planted at one mountain. Students can move trees from one mountain to another, or from one position of a mountain to another position of the same or different mountain. After making final selections, students click an on-screen “Submit” button to initiate the experiment. Then, the wind speeds before and after passing over each of the mountains with/without trees are shown on screen as experimental results.

This fair test has two variables: tree type (with three levels, corresponding to the three tree types) and tree position (with three levels, low, middle, and high). To conduct a fair test showing the effect of tree type, students must keep the tree positions across mountains identical. Table 2 shows the scoring rubric of the test. Since students can never plant the same type of trees on two mountains or at two positions of one mountain, the rubric focuses mainly on the types of trees planted on mountains. In addition, no matter how students plant trees, one mountain is left with no trees. A complete comparison on the effect of tree type needs a baseline condition of no trees, but students are not required to explicitly set up this condition in this test. Therefore, although there are in principle 3 × 3 × 3 × *P*(4,3) = 648 ways of tree planting and 3 × *P*(4,3) = 72 in which match the fair test requirement (*P* means permutation), the matching answers can be classified into three types: (a) those having the three types of trees all planted at the “Low” positions of any three out of the four mountains; (b) all at the “Middle” positions; and (c) all at the “High” positions. These answers receive a full score (3). Answers having trees planted at two distinct positions of any three mountains has a partial score (2), and those having trees planted at three distinct positions of any three mountains receives the lowest score (1).

**Table 2:** Scoring rubrics of the fair tests 1 and 2.

Score	Rubric of the fair test 1	Rubric of the fair test 2
3	Trees are planted at the same positions of three mountains (e.g., Low; Low; Low in Figure 2A)	Select three distinct ingredients with identical amount (e.g., Figure 3B)
2	Two types of trees are planted at the same positions of the mountains (e.g., Medium; High; High in Figure 2B)	Select three distinct ingredients, but two of them have identical amounts or all three have distinct amounts (e.g., Figure 3C)
1	Tree positions on the mountains are distinct (e.g., Low; High; Medium in Figure 2C)	None of the above (e.g., Figure 3D)

**Figure 3:**
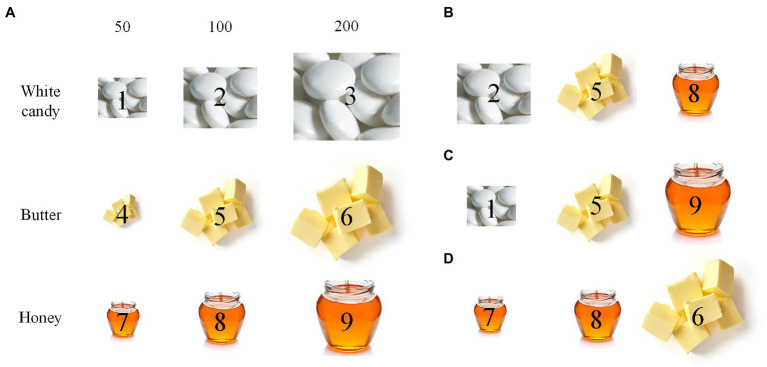
Example answers of the fair test 2. **(A)** Nine piles of ingredients for selection: 50, 100, and 200 are milligrams, and each pile is marked by an index of 1–9. **(B)** A choice of three piles, denoted by 2–5–8, matching the fair test requirement. **(C)** A choice of three piles, 1–5–9, partially matching the requirement. **(D)** A choice of three piles, 7–8–6, not matching the requirement.

The fair test 2 comes from a physical science task. Its cover task is as follows. A bakery shop is developing a new product. The bakers want to test which of the three ingredients (white candy, butter, and honey) has the most acceptable sweetness in the new product. Each ingredient has three amounts to choose: 50, 100, and 200 milligrams. After instruction scenes, in the fair test scene, nine piles of the three ingredients with the three amounts are shown on the left side of the screen (see Figure 3A), and students can drag three of these piles into the three slots on the right side of the screen to show the effect of ingredients on the sweetness of the product. Students can move the piles from one slot to another. After making final choices, students click on an on-screen “Submit” button to initiate the experiment, and the sweetness of each choice is shown on the screen.

This fair test has two variables: ingredient type (white candy, butter, and honey) and ingredient amount (50, 100, and 200 milligrams). To show the effect of ingredients, one needs to keep ingredient amount identical across conditions. Among a total of *C*(9,3) × *P*(3,3) = 504 choices of three piles of ingredients (*C* means combination), 3 × *P*(3,3) = 18 match the fair test requirement. Table 2 shows the scoring rubric of the test. Answers matching the fair test requirement receive a full score (3), and others receive a partial (2) or the lowest score (1).

### 3.2. The exhaustive tests and scoring rubric

**Figure 4:**
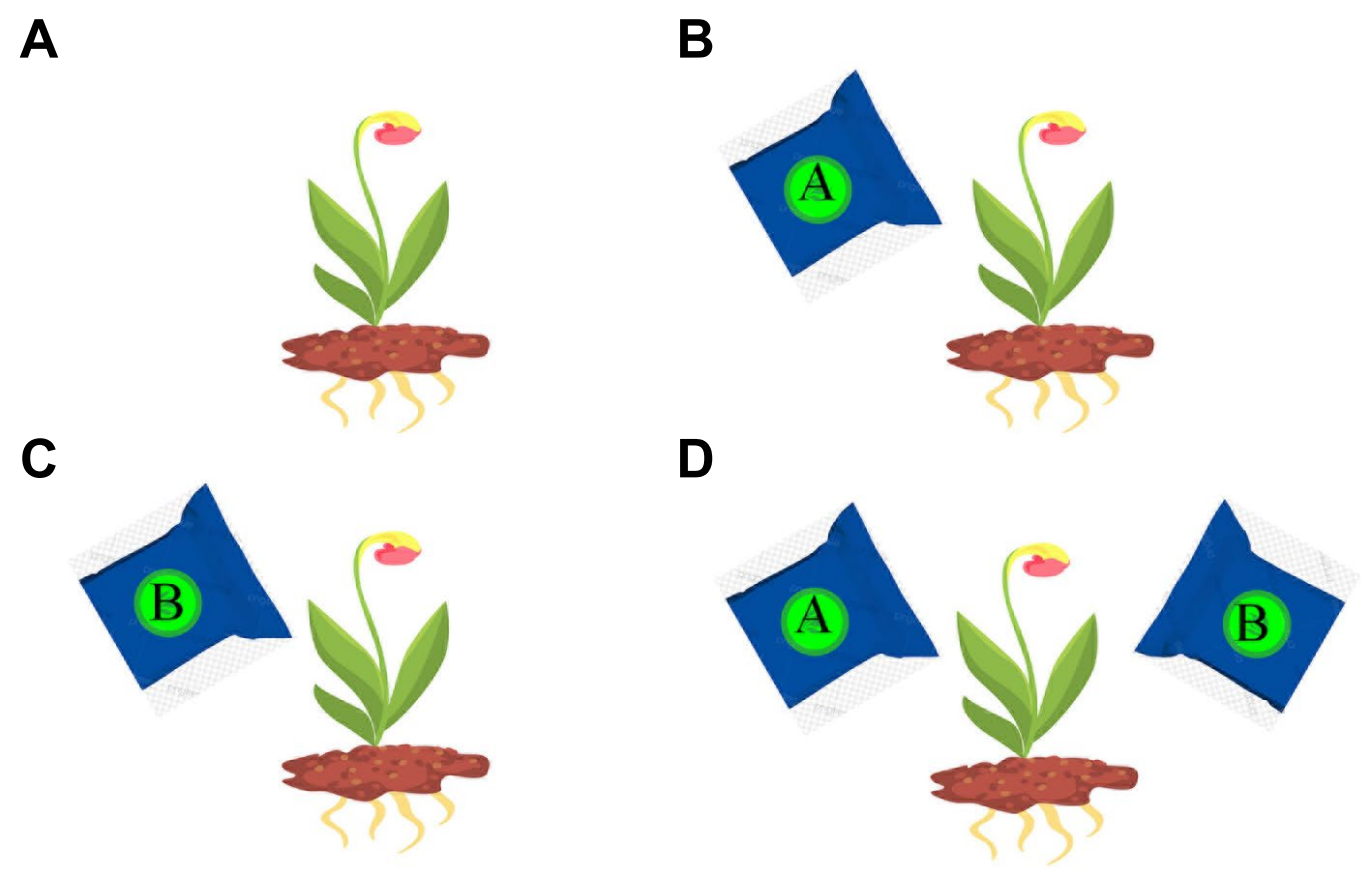
All the combinations in the exhaustive test 1: **(A)** None; **(B)**: A; **(C)**: B; **(D)**: A + B.

The exhaustive test 1 comes from a life science task. Its cover task is as follows. Farmers are trying to cultivate flowers with a special color. They do this in a natural way or using one or two types of fertilizers (A and B). After scenes for students to understand related issues, represent and explore different conditions, there comes the exhaustive test scene, in which students are asked to design an experiment to show which way has the highest chance to cultivate flowers with a target color. They can set up a condition by selecting (or not) any (or both) fertilizer, and save it by clicking an on-screen “Save” button. They can also remove a saved condition by clicking it and an on-screen “Delete” button. After saving some conditions, they can click on an on-screen “Submit” button to submit all the saved conditions at that moment as final answers. This test requires four variable combinations (see Figure 4). The follow-up scenes ask students to review their answers and apply their knowledge in similar domains. Students went through these scenes in the same order and could not freely jump around the scenes.

Table 3 shows the scoring rubric of this test. The four scales are based on the types of the saved answers, especially whether they include some hard-to-foresee ones (e.g., Figures 4A,D). An exhaustive answer covering all combinations in Figure 4 receives the full score (4), whereas answers lacking one, two, or three of the combinations receive lower scores 3, 2, and 1. The validity of the rubric (whether it can reasonably reflect students’ intuitive conceptions and clarify students with various levels of problem solving skills) is beyond the scope of this paper.

**Table 3:** Scoring rubrics of the exhaustive tests 1 and 2.

Score	Rubric of exhaustive test 1	Rubric of exhaustive test 2
4	Answers cover all the four conditions: None, A, B, A + B.	The 15 chosen loc. Include: (1) One loc. in each of the 13 regions (except region 6). (2) One additional loc. in one of adjacent regions to City A. (3) One additional loc. in one of adjacent regions to City B.
3	Answers exclude None, OR exclude A or B.	At least 14 chosen loc. Match cases (1) and (2), or (1) and (3). At least 13 chosen loc. Match case (1) only.
2	Answers exclude A + B, OR exclude A + B and A or B, OR exclude None and A + B.	At least 2 loc. Match cases (2) and (3) above.
1	None of the above.	None of the above.

For the exhaustive test 1, the original rubric also evaluates whether students give a proper interpretation of submitted answers. Here, students are rescored based only on their saved conditions. “Loc.” stands for location.

**Figure 5:**
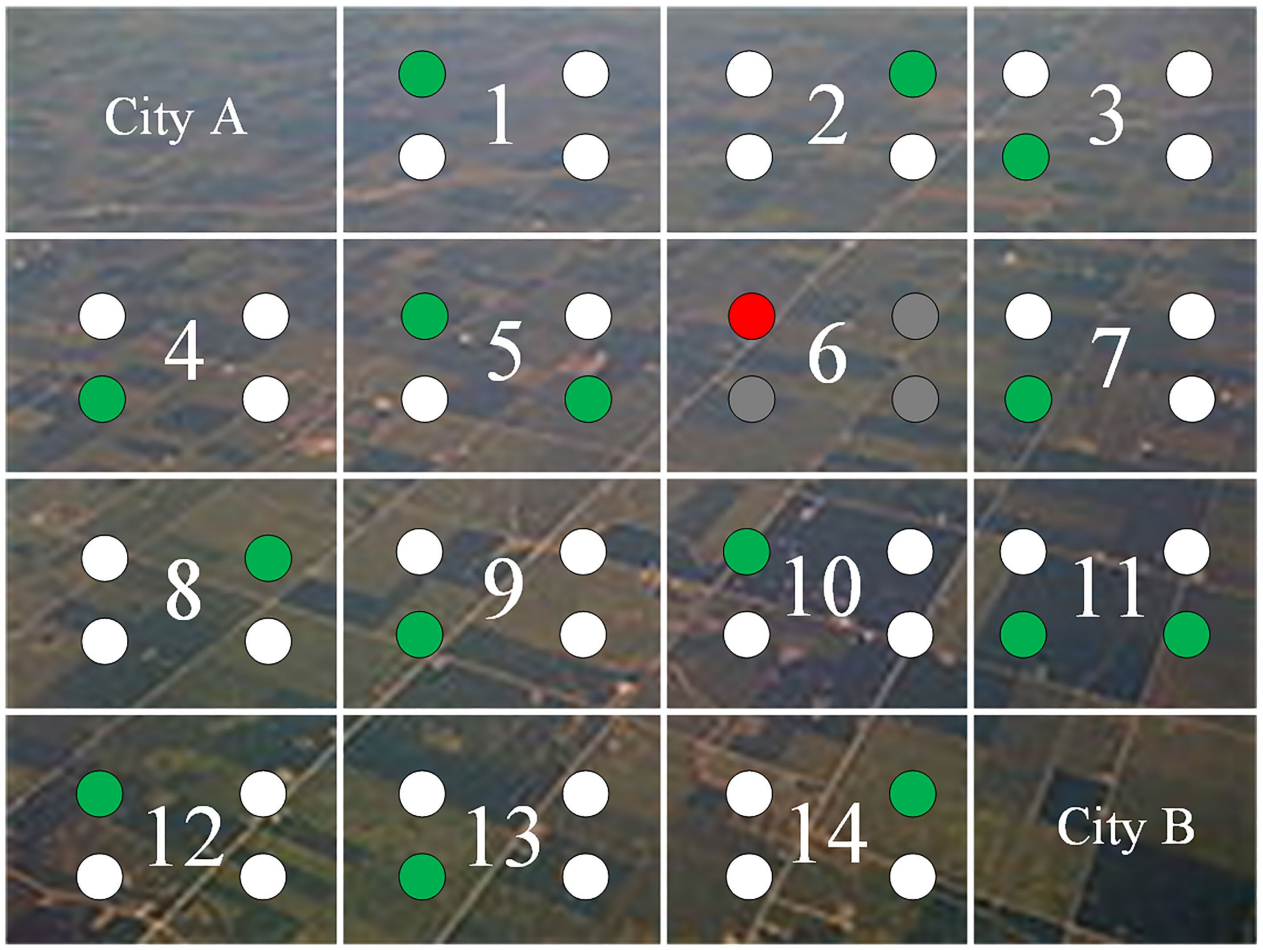
Example answers in the exhaustive test 2. Squares marked as 1–14 are the regions between City A and City B. Round dots in a region are locations for sample taking. Region 6 is the demo region with a chosen location marked in red, and the others are marked in grey. Green dots are students’ chosen locations. In this answer, there is at least one chosen location in all 13 regions except region 6, and there is at least one additional chosen location in one of the three regions adjacent to City A (region 5) and City B (region 11). This answer has a score of 4 (see Table 3).

The exhaustive test 2 comes from an earth science task. Its cover task is as follows. Two cities (A and B) plan to build a transmission tower to broadcast television signals. To evaluate signal quality on the land between the cities, they segment the land into 14 regions, each having four locations for signal sampling (see Figure 5). After instructions, students are asked to select at most 15 locations (out of 42) in the 13 regions (one region with one location therein being chosen is used as a demo) to test the signal coverage. They can select a location by clicking on it and deselect it by clicking on it again. If 15 locations are already chosen, students must deselect some chosen locations before making new selection(s). After choosing some locations (not necessarily 15), students can click on an on-screen “Submit” button to submit the chosen locations as final answers.

In this exhaustive test, students need to: (a) select at least one location in each of the 13 regions to test signal quality; and (b) choose two additional locations, respectively, in the three regions adjacent to each city to evaluate the signal sources in the two cities. It is challenging to foresee both aspects of requirements. Table 3 shows the scoring rubric of the test. The four scales are dependent on whether students fulfil both, either, or none of the two aspects of requirement. Whether this rubric is valid is not the focus of this paper.

### 3.3. Process-based measures

We define and measure three temporal features: preparation time (*PT*), execution time (*ET*), and mean execution time per answer-related event (*MET*). All of them are calculated based on time stamps of answer-related events. In the fair tests, answer-related events include: drag a type of trees (or a pile of ingredients) and drop it on a position of a virtual mountain (or a slot), or move a type of tree (or a pile of ingredients) from one mountain (position) (or one slot) to another; in the exhaustive tests, such events include: select one or two fertilizers (or a number of locations), and save or cancel a condition. The ending time point of *ET* is not the moment when students click on the “Submit” button, because after executing the last answer-related event, they can review their answers, thus moving into the next stage of problem solving. Also, executing actions may involve planning bounded to prior actions, which is different from the conceptual planning of related actions before making any. Therefore, we limited *ET* as the duration between the first and last answer related events. In addition to answer-related events, other factors (e.g., mouse or computer speed) might affect the efficiency of action execution. Since the tests were administered on site using the same model of tablets, the influence of these factors was minimal.

### 3.4. Preprocessing and analysis plan

Before analysis, we first remove missing values. Then, for each process feature in a data set, we adopt a 98% winsorization estimation (Dixon, 1960) (set the values <1% of the whole values to the value at 1%, and those >99% to the value at 99%) to adjust spurious outliers. Winsorization is independent of data distribution and preserves the exact proportion of data points, thus being more flexible than other outlier removal methods that presume a normal distribution of data points.

For response data, we first show score distributions among students and summarize how many students appropriately applied the CVS in each test, and then show the most frequent (top 10) submitted answers.

For process data, we conduct the Kruskal–Wallis test to compare the duration features across score groups. If a significant value of *p* is reported by the test, we adopt another non-parametric test, the Wilcoxon signed-rank test, on pair-wised score groups to clarify which pair(s) of score groups have different means of the features. These two tests, implemented using kruskal.test and wilcox.test functions in the *stats* package in R 3.6.1 (R Core Team, 2019), provide quantitative evidence on the relation between item scores and process features. Since there are three Kruskal–Wallis tests on the three measures, respectively, the critical *p-*value for identifying significance is set to 0.05/3 ≈ 0.0167.

To cross-validate the results of the Kurskal–Wallis and Wilcoxon signed-rank tests, we also conduct the omnibus ANOVA and pair-wised *t* tests (if the omnibus ANOVA test reports a significant value of *p*) between score groups. The log-transformed (base *e*) features pass the normality test (we use the Shapairo–Wilk’s method to test normality, and the *p*-values are all above 0.05, indicating that the distributions of the log-transformed data are not significantly distinct from a normal distribution). The ANOVA results are shown in the Supplementary materials.

## 4. Results

### 4.1. The fair tests

The two fair tests show similar trends in score distribution and top 10 frequent submitted answers.

**Figure 6:**
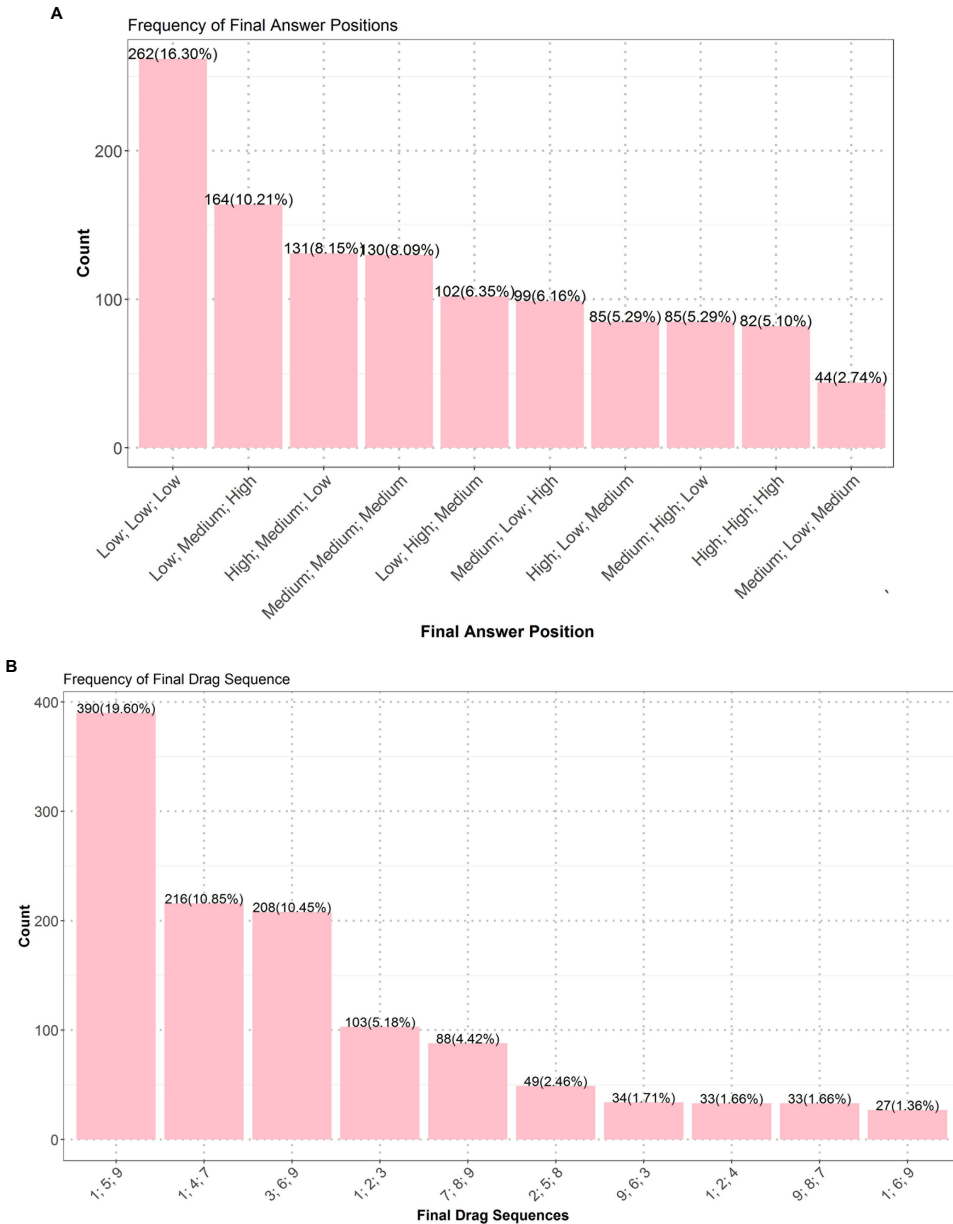
Top 10 frequent answers of the fair test 1 **(A)** and those of the fair test 2 **(B)**. Values on top of the bars are numbers of students, and those inside brackets are proportions.

In the fair test 1, 41.4% of the students received the lowest score (1), 29.1% received a partial score (2), and only 29.5% properly applied the CVS and got a full score (3). In other words, the majority (over 70%) of the students failed to properly apply the CVS in this test. Figure 6A illustrates the top 10 frequently-submitted answers in this test. “Low; Low; Low” was the most frequent correct answer, and other correct ones (e.g., “Medium; Medium; Medium” and “High; High; High”) were less frequent; “Low; Medium; High,” an answer with totally-varied tree positions, was the most common incorrect answer, and its variants (e.g., “High; Medium; Low” or “Low; High; Medium”) were also common, all receiving the lowest score (1); and the answers having a partial score (2) (e.g., “Medium; Low; Medium”) were less frequent.

In the fair test 2, only 28.8% of the students properly applied the CVS and got the full score (3), and most students had either the lowest score (1) (33.3%) or the partial score (2) (37.9%). Figure 6B shows that “1,4,7” was the most frequent correct answer, so was “3,6,9,” but other variants (e.g., “2,5,8” and “9,6,3”) were less frequent. “1,5,9,” an answer with totally varied ingredient amounts, was the most frequent incorrect answer. Others (e.g., “1,2,3,” “7,8,9” or “9,8,7”) that kept ingredient type consistent but varied ingredient amount were also frequent. Students who submitted these answers applied the CVS on a wrong variable. Other answers (e.g., “1,2,4” or “1,6,9”) that partially controlled the target variable of ingredient amount could not get the full score.

Table 4 shows the means and standard errors of the process features across score groups. As for the fair test 1, the Kruskal–Wallis tests report significant differences of these features across score groups (*PT*, *χ*^2^ = 12.2, df = 2, *p* < 0.005; *ET*, *χ*^2^ = 89.916, df = 2, *p* < 0.001; *MET*, *χ*^2^ = 64.776, df = 2, *p* < 0.001). The omnibus ANOVA tests show similar results [*PT*, *F*(2,1604) = 5.943, *p* < 0.005; *ET*, *F*(2,1604) = 51.7, *p* < 0.001; *MET*, *F*(2,1604) = 38.93, *p* < 0.001].

**Table 4:** Means and standard errors of *PT*, *ET*, and *MET* in each score group of the two fair tests.

Score	Fair test 1			Fair test 2		
	PT	ET	MET	PT	ET	MET
1	85.571 (1.166)	41.330 (1.098)	5.125 (0.091)	22.247 (1.007)	53.043 (1.650)	6.178 (0.170)
2	85.154 (1.407)	38.807 (1.216)	4.958 (0.103)	22.219 (0.832)	41.677 (1.265)	5.777 (0.134)
3	79.745 (1.172)	29.082 (1.081)	4.138 (0.090)	21.959 (0.874)	37.108 (1.387)	5.549 (0.157)

Values (in seconds) outside brackets are means and those inside brackets are standard errors.

As for the fair test 2, the Kruskal–Wallis tests report marginally significant differences in *PT* (*χ*^2^ = 7.824, df = 2, *p* = 0.02) and *MET* (*χ*^2^ = 6.600, df = 2, *p* = 0.037) and significant differences in *ET* (*χ*^2^ = 78.111, df = 2, *p* < 0.001) between score groups. The omnibus ANOVA tests show non-significant results for *PT* [*F*(2,1987) = 2.744, *p* = 0.065], but significant and marginally significant results for *ET* [*F*(2,1987) = 37.53, *p* < 0.001] and *MET* [*F*(2,1987) = 3.451, *p* = 0.032].

Table 5 shows the Wilcoxon signed-rank test results. In both fair tests, the students with higher scores had shorter *ET* than those with lower scores; and the full score students had shorter *PT* and *MET* than the lowest score students, but such differences were not statistically significant when the partial score group was involved.

**Table 5:** Wilcoxon signed-rank test results between pair-wised score groups of the two fair tests.

	Fair test 1			Fair test 2		
	PT	ET	MET	PT	ET	MET
1v2	158,942 (0.527)	**163,023 (0.016)**	158,766 (0.548)	229,631 (0.025)	**289,097 (0.001)**	252,368.5 (0.458)
1v3	**176,639 (<0.001)**	**2,038,350.5 (<0.001)**	**199,945.5 (<0.001)**	**177,693 (0.011)**	**247,740 (<0.001)**	**209,433.5 (0.014)**
2v3	**120,966.5 (0.014)**	**139,637.5 (< 0.001)**	**136,592.5 (< 0.001)**	212,462 (0.580)	**244,581.5 (<0.001)**	229,515 (0.056)

“1” to “3” refer to score groups. Values outside brackets are test statistics, and those inside are *p* values. Significant (having *p* values <0.0167) results are marked in bold. Supplementary Table S1 shows the omnibus ANOVA and pair-wise *t-*tests results.

### 4.2. The exhaustive tests

The two exhaustive tests show similar results.

**Figure 7:**
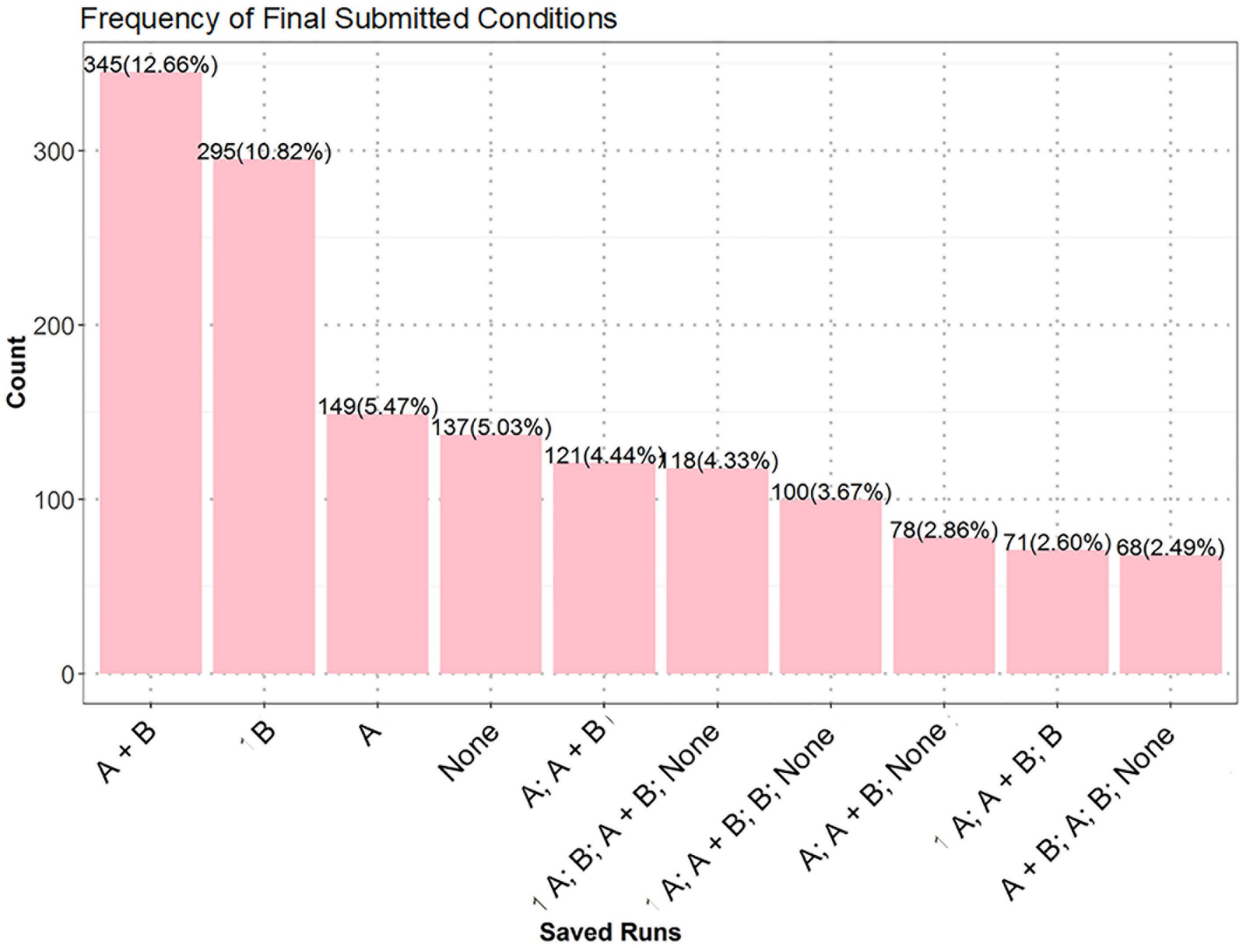
Top 10 frequent answers of the exhaustive test 1. Values on top of bars are numbers of students, and those inside brackets are percentages of students.

In the exhaustive test 1, 25.2% of the students received the lowest score (1), 33.9% properly applied the CVS and received the full score (4), and the rest got the partially high (3) (34.1%) or low (2) (6.8%) scores. In other words, the majority (over 65%) of the students failed to properly apply the CVS. Among the top 10 frequent answers (see Figure 7), “A; B; A + B; None” and its variants “A; A + B; B; None” and “A + B; A; B; None” received the full score, but they were less frequent than “A + B,” “B,” “A,” and “None,” which were the most frequent incorrect answers with the lowest score. Answers having partially high (e.g., “A; A + B; None”) or low (e.g., “A; A + B”) scores were less frequent.

In the exhaustive test 2, many students received the lowest score (1) (26.8%) and partially low score (2) (60.6%), and only 5.8% properly applied the CVS and got the full score (4), and 6% got the partially high score (3). Due to extremely numerous cases of submitted answers and equivalence of submitted answers, we discuss the frequent answers based on Figure 4 and the scoring rubric in Table 3. Most students got the partially low score (1), their submitted answers did not ensure that at least one location in each of the 13 regions was chosen; instead, they chose over 2 locations in the three regions adjacent to City A/City B, indicating that they failed to figure out the two requirements (see Section 3.2) of this test.

Table 6 shows the means and standard errors of the process features across score groups. In the exhaustive test 1, the Kruskal–Wallis tests report significant feature differences across score groups (*PT*, *χ*^2^ = 133.57, df = 3, *p* < 0.001; *ET*, *χ*^2^ = 498.49, df = 3, *p* < 0.001; *MET*, *χ*^2^ = 258.97, df = 3, *p* < 0.001). The omnibus ANOVA tests show similar results [*PT*, *F*(3,2721) = 65.4, *p* < 0.001; *ET*, *F*(3,2721) = 224.5, *p* < 0.001; *MET*, *F*(3,2721) = 78.4, *p* < 0.001].

**Table 6:** Means and standard errors of *PT*, *ET*, and *MET* across the score groups of the exhaustive tests 1 and 2.

Score	Exhaustive test 1			Exhaustive test 2		
	PT	ET	MET	PT	ET	MET
1	9.056 (0.325)	24.949 (0.922)	5.502 (0.144)	15.994 (0.388)	29.560 (0.670)	2.324 (0.043)
2	6.797 (0.439)	41.623 (1.804)	3.520 (0.113)	14.967 (0.231)	35.772 (0.473)	1.969 (0.022)
3	7.105 (0.207)	31.700 (0.715)	3.899 (0.070)	15.821 (0.705)	50.940 (1.624)	2.705 (0.076)
4	5.714 (0.172)	42.523 (0.763)	3.140 (0.051)	16.641 (0.755)	71.503 (2.053)	2.423 (0.066)

Values (in seconds) outside brackets are means and those inside are standard errors.

In the exhaustive test 2, the Kruskal–Wallis tests report marginally significant difference in *PT* (*χ*^2^ = 10.317, df = 3, *p* < 0.017), and significantly differences in *ET* (*χ*^2^ = 440.33, df = 3, *p* < 0.001) and *MET* (*χ*^2^ = 158.79, df = 3, *p* < 0.001). The omnibus ANOVA tests also show (marginally) significant differences in *PT* [*F*(3,2942) = 3.094, *p* = 0.026], *ET* [*F*(3,2942) = 185.7, *p* < 0.001], and *MET* [*F*(3,2942) = 45.58, *p* < 0.001].

Table 7 shows the Wilcoxon signed-rank test results. Similar to the fair tests, the full score students had shorter *PT* and *MET* than other low score students; but unlike the fair tests, the full score students had longer *ET* than most of the other score students. The patterns might not be consistent when partial score groups were involved.

**Table 7:** Wilcoxon signed-rank test results between pair-wised score groups of the two exhaustive tests.

	Exhaustive test 1			Exhaustive test 2		
	PT	ET	MET	PT	ET	MET
1v2	**750,18.0 (<0.001)**	**29,065 (<0.001)**	**83,948 (<0.001)**	743358.5 (0.034)	**547,881.5 (<0.001)**	**834,208 (<0.001)**
1v3	**372,475.5 (<0.001)**	**215,673.5 (<0.001)**	**400,288.5 (<0.001)**	77,802 (0.975)	**31,302 (<0.001)**	**60441.5 (<0.001)**
1v4	**422,941.5 (<0.001)**	**128,978.5 (<0.001)**	**458,813.5 (<0.001)**	63,511.5 (0.172)	**12,317 (<0.001)**	**60,717 (<0.001)**
2v3	84,443.5 (0.693)	**111,656 (<0.001)**	80,219.5 (0.147)	166,080.5 (0.197)	**100,023 (<0.001)**	**99,775 (<0.001)**
2v4	**98,433.5 (<0.005)**	81,207 (0.284)	**100,851 (<0.001)**	**135,210 (0.009)**	**41,858 (<0.001)**	**102,545 (<0.001)**
3v4	**501,936.5 (<0.001)**	**274,362.5 (<0.001)**	**531,023.5 (<0.001)**	15,784.5 (0.258)	**9,378 (<0.001)**	**19,416.5 (0.016)**

“1” to “4” refer to score groups. Values outside brackets are test statistics, and those inside are *p-*values. Significant (having *p-*values <0.0167) results are marked in bold. Supplementary Table S2 shows the omnibus ANOVA and pair-wise *t-*tests results.

## 5. Discussions

### 5.1. Problem solving processes of high- and low-performing students

This study examined two fair tests and two exhaustive tests from the NAEP scientific inquiry tasks, which require students to apply the control-of-variables strategy to design controlled experiments. We propose three process features to reflect the major stages of problem solving and use them to investigate performances of students having various levels of problem solving competency. In both types of tests, high- and low-performing students exhibited distinct response and process patterns.

In terms of response, more than 70% of the fourth- and eighth-graders failed to properly apply the control-of-variables strategy in the fair tests, and over 80% of the twelfth-graders failed to do so in the exhaustive tests. These are consistent with previous literature (Chen and Klahr, 1999).

In the fair test 1, the most common strategy was to vary tree position in mountain, e.g., “Low; Medium; High” (and its variations) (see Figure 6A). In the fair test 2, the most common strategy was to vary ingredient amount, e.g., “1,5,9” (and its variations) (see Figure 6B). These similar results are in line with early observations in response data (e.g., Shimoda et al., 2002): students adopting inappropriate strategies failed to recognize that variation in this extraneous variable actually interfered the effect of the target variable.

In the exhaustive test 1, the common wrong strategies were to save (and submit) only one of the four possible conditions. In the exhaustive test 2, the common wrong strategies were to select locations mainly in the regions adjacent to a city but ignore those in between. These inappropriate strategies reveal that: the low-performing students in these tests failed to conceive an exhaustive set of test data for the controlled experiments, probably due to lacking intention or required skills, and as a consequence, they simply submitted a subset of test data or some guessed answers. These results are in line with early studies (Tschirgi, 1980).

In terms of process, consistent patterns are evident in the process features. As for preparation time, in the fair tests, compared to students with the lowest score, those with a full score tended to spend shorter preparation time before making their first answer-related action. Longer preparation in students with the lowest score indicates that they needed more time to understand the test and plan their activities, whereas high-performing students could efficiently do so. This difference at the planning stage reveals that whether a student can properly solve a problem depends on whether he/she efficiently grasps the instructions and plans the activities *before* any is made. Apparently contradictive to the intuition that longer planning leads to better outcome, our finding is supported by results from other time-constrained tasks, e.g., a shorter pre-writing pause (duration between the moment a student entered the item and when he/she made the first typing event) in high-performing students in a time-constrained writing test, indicating efficient task planning (Zhang et al., 2017).

Patterns in preparation time between the high- and low-performing students were not consistent in the exhaustive tests. In the exhaustive test 1, students with the full score spent less preparation time than those with the lowest score, but in the exhaustive test 2, such pattern disappeared. The number of exhaustive combinations in the exhaustive test 1 (4) is much smaller than that in the exhaustive test 2 (15). Therefore, in the exhaustive test 2, both students with lower scores and those with the full score might not be able to foresee all required combinations at the planning stage, so they simply started right away to make selections and think along with the process of answer formation. This leads to non-significant difference in preparation time between the high- and low-performing students in this test.

As for execution time and mean execution time, in the two fair tests, most students with the lowest score spent longer execution time in conducting the drag-and-drop actions than those with higher scores (see Tables 4, 5). In these tests, the minimum number of actions required to construct an answer was just 3: drag and drop each type of trees (or three piles of different ingredients) respectively at the same (or different) positions of three mountains (or the three slots). There were two situations that caused longer execution time in students with the lowest scores: they either spent more time in executing individual actions or kept revising their choices,^7^ both reflecting hesitation or uncertainty during the action execution stage of problem solving. The process feature of mean execution time (see Tables 4, 5) explicitly reveals that on average, students with the lowest score spent more time on conducting each of their answer-related actions, i.e., they were less efficient in action execution than those with the full score.

Unlike the fair tests, in the exhaustive tests, most students with the lowest score showed shorter execution time than those with higher scores in answer formulation (see Tables 6, 7). According to Table 3, low scores in these tests correspond to incomplete submissions. The longest execution time of most full score students suggests that they were well motivated and had endeavored in constructing and saving all possible conditions, even at the cost of spending more time in total. By contrast, the shorter execution times of the lower score students were mostly caused by two cases: (1) they did not spend much time exploring the conditions and finished the test with lack-of-thinking results, which reflected low motivation/engagement or lack of reasonable understanding; (2) without realizing that they needed to submit all possible conditions, some students left the test after submitting just one condition (consistent with the frequent wrong answers).

As for mean execution time, in the exhaustive test 1, though spending more time in problem solving, most students with the full score showed shorter mean execution time than those with the lower scores (see Tables 5, 6). This indicates that most of high-performing students efficiently formulated their answers. In the exhaustive test 2, although spending longer time in selecting multiple locations for comparison, most high-performing students had smaller or comparable mean execution time to that of low-performing students, who submitted incomplete answers. To sum up, in both tests, high-performing students tended to be more efficient in executing multiple answer-related actions than low-performing ones.

### 5.2. Process features and problem-solving competency

In all four tests, most students who properly applied the control-of-variables strategy (thus having high problem-solving competency) enacted more goal-oriented behaviors (Shimoda et al., 2002). In the fair tests, they quickly grasped the goal at the planning stage, and efficiently set up the conditions matching the fair test requirement; in the exhaustive tests, with a clear goal in mind, they persistently constructed all the conditions for comparison within a longer execution time. By contrast, students having low problem-solving competency were confused about the target variable while formulating answers in the fair tests; in the exhaustive tests, they either ignored or did not fully understand the goal, and tended to drop before submitting enough conditions.

The proposed process features of execution time and mean execution time reflect the level differences in goal-orientation and motivation between students, which are crucial to problem solving (Gardner, 2006; Dörner and Güss, 2013; Güss et al., 2017). The contrasting patterns of execution time between the two types of tests reveal different characteristics of the solutions and execution stages therein; the fair tests need conditions matching the fair test requirement, yet the exhaustive tests request all possible conditions. They also reveal that task property could influence how students deploy strategies that they seem to know, which echoes the knowledge-practice integration in NGSS.

The consistent patterns of mean execution time in high-performing students across the two types of tests indicate that both types of tests require similar control-of-variables strategies and high-performing students can efficiently apply such strategies in solving apparently-distinct problems. This suggests that the capabilities of doing analogical reasoning and employing key skills and related abilities across tasks of various contents are critical in scientific problem solving.

Most of the above discussions concern the full and lowest score students, because the statistical tests report consistent results between these groups in each test. Inconsistent results exist between partial score groups or between a partial score group and the full (or the lowest) score group. This inconsistency is due to several reasons. First, some partial score groups contained fewer students than others. Second, as in the scoring rubrics, the response difference between the full (or the lowest) and a partial score is smaller than that between the full and the lowest scores. Both of these factors decimated the statistical power of the analyses. Third, due to lacking empirical bases (Parshall and Brunner, 2017), the predefined rubrics might not be able to clearly differentiate students with different levels of problem solving competency. The reliability of scoring rubrics is worth further investigation, but it is beyond the scope of the current study.

The discussions on problem solving process and competency based on process features of high- and low-performing students in different tests provide useful insights on teaching and learning of the control-of-variables strategies and related skills as well as applying them in similar scientific inquiry tasks. For example, comparing a specific student’s performance with the typical patterns of high-performing students can reveal on which problem solving stage the student needs to improve efficiency; comparing high- and low-performing students’ process patterns can also reveal on which aspects the low-performing students need to polish, e.g., how to allocate time and effort in different problem-solving stages in order to improve overall performance in scientific inquiry tasks.

### 5.3. Precision of process features

The temporal features of preparation time and execution time roughly estimate the process of action planning and that of action execution, respectively. In addition to individual differences, other factors may “contaminate” these features, especially in complex tasks requiring careful thinking and multiple answer formulation stages; e.g., students may change part of their answers during the problem solving process, and execution time may cover the time of answer change.

Answer change is part of action execution. In all four tests, most students conducted answer change through drag-and-drop actions. For example, in the fair test 1, the minimum number of drag-and-drop actions for correctly answering the question is 3, but only 11% of the students conducted exactly 3 drag-and-drop actions, and more than 50% conducted 3 to 6 actions; in the exhaustive test 1, the minimum number of saved conditions for a correct answer is 4, but only 23% of the students saved exactly 4 cases, and more than 90% saved 4–6 cases. In addition, answer change actions are often intertwined with answer formulation actions, indicating that the purpose of such actions is to correct execution error and stick to planned actions. In this sense, answer change is part of action execution, and their durations should be included into execution time.

However, students might occasionally clear all the answers and re-answer the question from scratch. In this case, they could spend some time to re-plan their actions, but such time is embedded in the current definition of execution time. In the four tests, very few (<1%) students went through such re-planning and re-execution process, but in complex tasks, such cases may be ample. To better clarify such cases, we need to improve the precision of process features by examining drag-and-drop action sequences and their time stamps to clearly identify whether a student re-planned. We leave such modification to future work.

### 5.4. Procedure of process data use

In addition to the process features and insights on scientific problem solving, this study lays out a general procedure of using process data to study test-takers’ performance or competency:

*Discover or define process features that could (potentially) inform test-takers’ performance or competency*. This step is often based on prior hypotheses or existing studies;

*Demonstrate correlation or relatedness between process features and test-takers’ performance*. This step is critical in two aspects. First, it verifies whether the features are related to performance in the target dataset. Second, it bridges the first and third steps; only after relatedness or correlation between test-takers’ performance and the process features is validated would analyses on these features and derived understandings become meaningful.

*Understand or characterize test-takers’ performance, or incorporate process features into scoring rubrics, cognitive or measurement models*. Understanding test-takers’ performance is based on defined or discovered features in the first step. In our study, the proposed features characterize high- and low-performing (or common vs. abnormal) test-takers. The observed consistent patterns of process features also pave the way for incorporating those features into scoring rubrics, e.g., specific values or ranges of values of process features correspond to various scales of scores. Moreover, the quantitative process features as in our study could serve as important components in cognitive or measurement models to predict, classify, or interpret test-takers’ performance.

## 6. Conclusion

This study proposes three process features and an analytical procedure of process data use. Based on four scientific inquiry tasks, we investigate how students apply the control-of-variables strategy in typical fair and exhaustive tests and how the process features characterize high- and low-performing students in these tasks. Although (meta)cognitive processes cannot be observed directly from process data, the proposed features have proven values in elucidating the planning and executing stage of problem solving, characterizing students’ performance patterns, and revealing relatedness among capacities (the control-of-variables strategy), test properties (the fair and exhaustive tests), and performance (answers, scores, and answering process). Our study demonstrates that process data provide unique windows to interpret students’ performance beyond scores, and that a combination of analytical procedures and process data helps infer students’ problem-solving strategies, fill in the gap in early studies, and stimulate future work on process features reflecting problem-solving performance.

## Data availability statement

The raw data supporting the conclusions of this article will be made available by the authors, without undue reservation.

## Ethics statement

The studies involving human participants were reviewed and approved by NCES. Written informed consent to participate in this study was provided by the participants’ legal guardian/next of kin.

## Author contributions

TG and LS designed the study. TG collected the data and conducted the analysis and wrote the manuscript. TG, LS, YJ, and BA discussed the results. LS, YJ, and BA edited the manuscript. All authors contributed to the article and approved the submitted version.

## Conflict of interest

TG was employed by the company Google.

The remaining authors declare that the research was conducted in the absence of any commercial or financial relationships that could be construed as a potential conflict of interest.

## Publisher’s note

All claims expressed in this article are solely those of the authors and do not necessarily represent those of their affiliated organizations, or those of the publisher, the editors and the reviewers. Any product that may be evaluated in this article, or claim that may be made by its manufacturer, is not guaranteed or endorsed by the publisher.

## Footnotes

^1^ https://nces.ed.gov/

^2^Such data include, but are not limited to: *student events and their time stamps*, e.g., drag-and-drop, (de)selection, or tool-use actions during answer formulation, text (re)typing or editing behaviors during keyboard-based writing, or navigations across pages, scenes, or questions during on-screen reading; and *system events and their time stamps*, e.g., entering/leaving scenes, (de)activating on-scene tools or popping-up messages.

^3^In addition to durations, one can measure numbers/sequence of answer-related events in the test scene. Such features have some uncertainties: greater/fewer events do not necessarily require more/less effort, more events sometimes indicate low competency, and it is non-trivial to align such features with performance (scores). The duration features proposed in our study can overcome these uncertainties by explicitly measuring students’ planning and executing stages and relating them to performance.

^4^Some recent studies begun to touch upon duration features. For example, Arslan et al. (2020) reported no significant correlations between item scores and preparation (and execution) times in math items. Jiang et al. (2021) investigated action sequences and response strategies derived. One can also measure ratios between durations. If durations can reflect the planning and executing stages during problem solving and characterize performance patterns of students, ratios between durations can further reveal relative cognitive loads between the planning and executing processes. We leave such ratio-based features in future work.

^5^*a.k.a.* "isolation of variables" (Inhelder and Piaget, 1958), "vary one thing at a time" (Tschirgi, 1980), or "control of variables strategy" (Chen and Klahr, 1999).

^6^Due to privacy and secure nature of NAEP data, we present conceptually equivalent cover tasks to maintain task security. The cover tasks have similar underlying structures and require similar cognitive processes to solve, but do not connect to specific science contents as the real tasks.

^7^Both can be identified from event logs, and action frequency data can further clarify which situation is more popular.
