# Supplementary material for: Using process features to investigate scientific problem-solving in large-scale assessments
Source: Front Psychol. 2023 Apr 18;14:1131019. doi: 10.3389/fpsyg.2023.1131019 (PMC10151488; doi:10.3389/fpsyg.2023.1131019)
Supplement: Supplementary file 1 [file Table_1.DOC]

Supplementary Materials

**Scientific Problem Solving and Scientific Inquiry**

*Problem solving* is a cognitive process directed at transforming a given situation into a goal situation when no obvious solution is available, and *problem solving competency* is an individual’s capacity to engage in understanding and resolving problem situations with no obvious method of solution (Mayer, 1990). Progressively developed in high quality education through problem/inquiry based learning and individual/group project, this competence is one of the most important skills in STEM education of the 21^st^ century (OECD, 2013).

Theoretical frameworks of problem solving have been discussed in educational psychology. Dewey (2007) proposes the core components of problem solving, including: perceived difficulty, problem definition, possible solutions/hypothesis, and tests of applied solutions. Polya (2007) updates this theory with a five-stage framework: formulating objectives; defining situation; planning, generating ideas, and choosing alternative solution(s); executing chosen alternative(s); and testing results.[[1]](#footnote-2) Some scholars focus on invariant or universal cognitive operations during problem solving, whereas others highlight processes particularly relevant to problem solving.[[2]](#footnote-3) Despite perspective differences, many theories state how problems are solved, rather than what problem solving is or how it is conceived, and most are based on response/survey data and limited at a conceptual level (Botía & Orozco, 2009). Among others, Mayer’s descriptive definitions (see the first paragraph of the section) have been widely accepted by the problem-solving community (Mayer & Wittrock, 1996; Reeff et al., 2006).

Large-scale assessments provide an empirical platform to evaluate problem solving. In 2003, the Program for International Student Assessment (PISA) launched by the Organization for Economic Co-operation and Development (OECD) initiated an operational framework to assess problem solving competency (OECD, 2004). NAEP developed a similar framework, in line with cognitive psychology (Mayer & Wittrock, 2006). This operational framework of problem solving consists of the following four stages:

(1) *Explore and understand*, to build mental representations of each information presented;

(2) *Represent and formulate*, to build a coherent mental representation of the problem;

(3) *Plan and execute*, to set up overall/sub-goals, devise a plan/strategy to reach the goal state, and carry out the plan by executing related strategies/actions; and

(4) *Monitor and reflect*, to check final/intermediate results, evaluate assumptions and alternative solutions, and look for additional information/clarification.

This framework has stimulated explorations in complex problem-solving and computer-based assessment (Blech & Funke, 2010; Funke, 2010; Wirth & Klieme, 2004), many lying in mathematics education and focusing on describing concept formation.

With the advancement in PISA/NAEP, scientific problem solving has become a key subject in problem solving research (OECD, 2013). Scientists and educators advocate teaching science as inquiry, rather than factual knowledge (Mislevy et al., 2003; Rönnebeck et al., 2016). *Scientific inquiry* refers to activities through which students develop knowledge of scientific ideas and how to investigate the natural world and solve problems in a scientific way (National Research Council, 1996). Such activities include: question identification, information search, hypothesis formulation, argumentation engagement, and so on (Scalise, 2014). They are an important goal of scientific literacy and a key component of scientific problem solving (Bybee, 2000). Among various activities, planning, designing, and carrying out controlled experiments correspond roughly to the planning and executing stage of the operational framework of problem solving, thus becoming a principal focus of scientific inquiry practice in children and youngsters (Chen & Klahr, 1999).

NAEP Science Tasks

NAEP is a congressionally mandated, nationwide digital assessment project administered by the National Center for Education Statistics (NCES). NAEP provides assessments on disciplines of math, reading, writing, science, social science, and others. Assessments are designed and updated regularly by content specialists, education experts, and teachers around the U.S. Participants of NAEP tests are fourth- (~9-year-olds), eighth- (~13-year-olds), and twelfth-graders (~17-year-olds) from different schools. Survey data of students, teachers, and schools are collected with assessment data, covering demographical and socio-economic information, language learning programs, etc. The NAEP samples have roughly-equal gender ratio and typical distributions of major ethnic groups in the U.S. NAEP has now become the largest and most important national assessments of what U.S. students know and can do.

The 2018 NAEP science pilot study was conducted in schools across the nation. Students were asked to finish two 30-minute tasks (blocks). Their performances were largely consistent across blocks, as indicated by high Cronbach’s alphas (> 0.85). A repertoire (32) of tasks were designed and administered, falling into physical, life, and earth/space science, in line with NGSS treating knowledge-practice integration as a key learning goal and a criterion of meaningful science learning (National Research Council, 2014). Test items in the blocks were scored by humans (e.g., constructed response items) and/or computers (e.g., single/multiple selection items). In particular, the D&D items were scored by computers using submitted answers and against predefined rubrics.

**Table S1. Omnibus ANOVA and pair-wise *t* tests results of the two fair tests. “1” to “3” are scores. Values outside brackets are *t* values, those inside are adjusted Tukey *p* values. Significant (having *p* values < .0167) results are marked in bold.**

|  | ***fair test 1*** | | | ***fair test 2*** | | |
| --- | --- | --- | --- | --- | --- | --- |
|  | ***PT*** | ***ET*** | ***MET*** | ***PT*** | ***ET*** | ***MET*** |
| 1v2 | .347 (.936) | **2.056 (.099)** | 1.518 (.283) | .367 (.929) | **5.137 (< .001)** | .971 (.595) |
| 1v3 | **3.266 (.003)** | **9.91 (< .001)** | **8.540 (< .001)** | .125 (.991) | **9.578 (< .001)** | **2.601 (.015)** |
| 2v3 | **2.689 (.020)** | **7.236 (< .001)** | **6.466 (< .001)** | .226 (.972) | **4.862 (< .001)** | 1.730 (.194) |

**Table S2. Omnibus ANOVA and pair-wise *t* tests results of the two exhaustive tests. “1” to “4” are scores. Values outside brackets are *t* values, those inside are adjusted Tukey *p* values. Significant (having *p* values < .0167) results are marked in bold.**

|  | ***exhaustive test 1*** | | | ***exhaustive test 2*** | | |
| --- | --- | --- | --- | --- | --- | --- |
|  | ***PT*** | ***ET*** | ***MET*** | ***PT*** | ***ET*** | ***MET*** |
| 1v2 | **4.350 (< .001)** | **-13.480 (< .001)** | **8.396 (< .001)** | 1.675 (.337) | **-10.107 (< .001)** | **6.124 (< .001)** |
| 1v3 | **6.265 (< .001)** | **-12.009 (< .001)** | **10.878 (< .001)** | -.590 (.935) | **-14.367 (< .001)** | **-5.712 (< .001)** |
| 1v4 | **11.982 (< .001)** | **-23.899 (< .001)** | **18.382 (< .001)** | -1.533 (.418) | **-21.125 (< .001)** | **-3.071 (.012)** |
| 2v3 | .560 (.575) | **6.365 (< .001)** | -1.839 (.066) | -1.578 (.391) | **-9.475 (< .001)** | **-9.542 (< .001)** |
| 2v4 | **3.022 (< .005)** | -1.089 (.276) | **2.867 (< .005)** | -2.511 (.058) | **-16.858 (< .001)** | **-6.513 (< .001)** |
| 3v4 | **6.212 (< .001)** | **-12.919 (< .001)** | **8.158 (< .001)** | -.786 (.861) | **-6.081 (< .001)** | 1.882 (.236) |

References

Blech, C. & Funke, J. 2010. You cannot have your cake and eat it, too: How induced goal conflicts affect complex problem solving. *Open Psychology Journal*, 3: 42–53.

Botía, M. L. & Orozco, L. H. 2009. Critical review of problem solving processes traditional theoretical models. *International Journal of Psychological Research*, 2(1), 67–72.

Bybee, R. W. 2000. Teaching science as inquiry. In: J. Minstrell & E. H. van Zee (Eds.), *Inquiring into inquiry learning and teaching in science*, Washington, DC: American Association for the Advancement of Science, pp. 21–46.

Chen, Z. & Klahr, D. 1999. All other things being equal: acquisition and transfer of the control-of-variables strategy. *Child Development*, 70(5): 1098–1120.

Dewey. J. 2007. The collected works of John Dewey. In: J. A. Boydston (Ed.), *John Dewey (1859–1952): The internet encyclopedia of philosophy* (Originally published in 1910). <http://www.utm.edu/research/iep/d/dewey.html>.

Funke, J. 2010. Complex problem solving: A case for complex cognition? *Cognitive Processing*, 11: 133–142.

Landau, E. 1987. *El vivir creativo*. Barcelona, Spain: Herder.

Linhart, J. 1976. *Činnost a poznávání*. Praha: Academia.

Mayer, R. E. 1990. Problem solving. In: W. M. Eysenck (Ed.), *The Blackwell dictionary of cognitive psychology*. Oxford: Basil Blackwell, pp. 284–288.

Mayer, R. E. & Wittrock, M. C. 1996. Problem solving transfer. In: R. Calfee & R. Berliner (Eds.), *Handbook of educational psychology*, New York: Macmillan, pp. 47–62.

Mayer, R. E. & Wittrock, M. C. 2006. Problem solving. In: P. A. Alexander & P. H. Winne (Eds.), *Handbook of educational psychology* (2^nd^ ed.). Mahwah, NJ: Lawrence Erlbaum Associates, pp. 287–304.

Mislevy, R. J., Chudowsky, N., Draney, K., Fried, R., Gaffney, T., et al. 2003. *Design patterns for assessing science inquiry (PADI Technical Report 1)*. Menlo Park, CA.

National Research Council. 1996. *National science education standards*. Washington, DC: National Academies Press.

National Research Council. 2014. *Developing assessments for the Next Generation Science Standards*. Washington, DC: National Academies Press.

OECD. 2004. *Problem solving for tomorrow’s world: First measures of cross-curricular competencies from PISA 2003*. Paris: OECD.

OECD. 2013. *PISA 2012 assessment and analytic framework: Mathematics, reading, science, problem solving and financial literacy*. Paris: OECD.

Polya, G. 2007. How to solve it. In: P. Alfeld (Ed.), *Understanding mathematics* (2nd ed.) (Originally published 1957). <http://www.math.utah.edu/~pa/math/polya.html>.

Reeff, J. P., Zabal, A., & Blech, C. 2006. *The assessment of problem-solving competencies: A draft version of a general framework*. Bonn, Germany: Deutsches Institut für Erwachsenbildung.

Rönnebeck, S., Bernholt, S., & Ropohl, M. 2016. Searching for a common ground – A literature review of empirical research on scientific inquiry activities. *Studies of Science Education*, 52(2): 161–197.

Scalise, K. 2014. *Assessment system design options for the next generation science standards (NGSS): Reflections on some possible design approaches*. Invitational Research Symposium on Science Assessment, Princeton, NJ.

Sternberg, R. 1980. Representation and process in linear syllogistic reasoning. *Journal of Experimental Psychology*, 109: 119–159.

Wirth, J. & Klieme, E. 2004. Computer-based assessment of problem solving competence. *Assessment in Education: Principles, Policy and Practice*, 10(3): 329–345.

1. Others propose different stages, e.g., Linhart’s three-stage (discovery of problematic situation, solution, and verification of discovered property/method) framework (1976); Landau’s three-stage (preparation, production, and judgment) framework (1987); and Sternberg’s cycling framework (1980), each cycle comprising repetitive steps of recognizing the problem, organizing related knowledge, developing a solving strategy, allocating resources, checking progress and testing adjustment to specific situation(s). [↑](#footnote-ref-2)
2. E.g., information processing (Botía & Orozco, 2009), personal aim (Mayer & Wittrock, 1996), creative or critical thinking (Mayer, 1998), and prior knowledge on the problem (Funke, 2010). [↑](#footnote-ref-3)
